# Mechanical and Thermal Properties of Epoxy Resin upon Addition of Low-Viscosity Modifier

**DOI:** 10.3390/polym16172403

**Published:** 2024-08-24

**Authors:** Yingnan Wang, Pierre Mertiny

**Affiliations:** Department of Mechanical Engineering, University of Alberta, Edmonton, AB T6G 1H9, Canada; yingnan1@ualberta.ca

**Keywords:** epoxy resin, low-viscosity modifier, rheology, curing kinetic, thermal properties, mechanical properties, morphology

## Abstract

Thermoset-based polymer composites containing functional fillers are promising materials for a variety of applications, such as in the aerospace and medical fields. However, the resin viscosity is often unsuitably high and thus impedes a successful filler dispersion in the matrix. This challenge can be overcome by incorporating suitable low-viscosity modifiers into the prepolymer. While modifiers can aptly influence the prepolymer rheology, they can also affect the prepolymer curing behavior and the mechanical and thermal properties of the resulting matrix material. Therefore, this study investigates the effects that a commercial-grade low-viscosity additive (butyl glycidyl ether) has on a common epoxy polymer system (diglycidyl ether of bisphenol-A epoxy with a methylene dianiline curative). The weight percentage of the modifier inside the epoxy was varied from 0 to 20%. The rheological properties and cure kinetics of the resulting materials were investigated. The prepolymer viscosity decreased by 97% with 20 wt% modifier content at room temperature. Upon curing, 20 wt% modifier addition reduced the exothermic peak temperature by 12% and prolonged the time to reach the peak by 60%. For cured material samples, physical and thermo-mechanical properties were characterized. A moderate reduction in glass transition temperature and an increase in elastic modulus was observed with 20 wt% modifier content (in the order of 10%). Based on these findings, the selected material system is seen as an expedient base for material design due to the ease of processing and material availability. The present study thus provides guidance to researchers developing polymer composites requiring reduced prepolymer viscosity for successful functional filler addition.

## 1. Introduction

Thermosetting polymer composites are widely used in the aerospace, automotive, sports, and other industries. Liquid composite molding (LCM) processes [1,2], such as resin transfer molding (RTM) [3,4,5] and vacuum-assisted resin infusion (VARI) [6,7,8], are recognized as highly adaptable, economic, and efficient fabrication methods for epoxy-based composite structural parts with demanding geometric complexity. However, despite these benefits, fabricating high-quality structures via LCM remains challenging work. In contrast to autoclave processes [9,10], low fiber volume and high porosity are two main drawbacks frequently associated with LCM. To date, significant research has been conducted to overcome these challenges. One approach is adding injection pressure or pressure after injection [11,12]; another approach is lowering the prepolymer viscosity to ease resin flow, which can be achieved by employing heating [13,14,15] and/or additives [16,17]. The latter can be solvents that are removed from the material during processing, or suitable modifiers that become part of the composite system. Incorporating modifiers is an attractive proposition as they are available in different physical states and chemical compositions, thus enabling the design of modified polymer matrices with tailored characteristics, including rheology, cure kinetics, and mechanical and physical properties.

The technical literature describes a broad range of modifiers and their influences on polymer systems. In [18], a silane-coupling agent (3-aminopropyltriethoxysilane) was used to modify an epoxy resin to improve the tensile properties of viscose fabric composites. The modifier’s effect on the prepolymer viscosity, the glass transition temperature, and the contact angle of cured castings were also evaluated. A different study [19] focused on rapid RTM processes, where a reactive diluent (1,4-butanediol) and catalyst (1-benzyl-2-methylimidazole) were used to modify an epoxy system. The effects on the liquid phase and gel time of the system were studied by rheological processes. A reactive modifier (HELOXY 61, Miller-Stephenson, Danbury, CT, USA) was used in [20] to lower viscosity along with a coupling agent to improve the adhesion property of epoxy formulations. In [21], poly(styrene-co-acrylonitrile), a thermoplastic modifier, was used to modify an epoxy resin system. Morphology, dynamic-mechanical, and thermal properties were characterized for modified epoxy resin/glass fiber composites. Similarly, the study described in [22] investigated the effects of a thermoplastic modifier, polyetherimide (PEI), on the relationship between phase morphology and fracture toughness of two modified epoxy monomers systems, i.e., diglycidyl ether of bisphenol A (DGEBA) and tetraglycidyl-4,4′-diaminodifenylmethaan. A strong increase in fracture toughness was noticed at the highest PEI content. Three modifiers were evaluated as potential tougheners for epoxy resin systems by Russell [23], namely, an epoxy-terminated aliphatic polyester hyperbranched polymer (EI), a carboxy-terminated butadiene rubber, and an aminopropyl-terminated siloxane. The results revealed that the EI modifier had the best balance of properties, significantly improving toughness while having a minimal effect on the flexural modulus, glass transition temperature, viscosity, and gelation time. Likewise, the study in [24] investigated four varieties of molecular weight and epoxy equivalent weight liquid thermosetting hyperbranched poly(trimellitic anhydride-diethylene glycol) ester epoxy resin as modifiers to enhance the toughness and mechanical properties of an epoxy resin system. The researchers in [25] selected benzyl methacrylate and poly(ethylene glycol) methyl ether methacrylate as modifier monomers for in situ radical polymerization of carbon fiber reinforced epoxy polymer composites, effectively enhancing the fracture toughness of the carbon fiber reinforced laminates while maintaining a low viscosity of the uncured resin system. In [26] the effects of an ABA-type commercial grade acrylic tri-block-copolymers on a modified epoxy resin system were demonstrated, showing improved toughness without significant reduction of desirable mechanical (e.g., stiffness and strength) and thermal (e.g., glass transition temperature) properties. The work described in [27] used both liquid additives (salt solution of unsaturated polyamine amides and acidic polyester, silicon rubber) and solid particles (layered silicates, SiO_2_ particles and carbon black) to a cycloaliphatic epoxy resin, with and without surface treatment. The cure kinetics of the system were investigated by isothermal and non-isothermal differential scanning calorimetry (DSC). The researchers in [28] investigated the effects of additional dimer acid diglycidyl ester (DAGE) on mechanical properties (e.g., impact strength, tensile strength, elongation), thermal properties (e.g., glass transition temperature, thermal stability), and curing kinetics of the epoxy resin. Rougher fracture surfaces were observed with higher DAGE contents, which were found to be accompanied by increasing impact strength.

From the preceding brief review of the technical literature, it becomes apparent that adding modifiers can influence the chemical structure of a polymer matrix by promoting or impeding the interaction of functional groups, while also affecting the rheological, mechanical, thermal, and other materials properties. Thermosetting polymer (epoxy-based) composites can be employed in diverse industrial applications, often necessitating the development of multifunctional composites. Therefore, a variety of modifiers can be chosen to achieve specific functions in modified composites. For the purpose of multifunctionality, low pre-polymer viscosity is typically a significant aspect, for example, to facilitate the incorporation of fillers such as solid particles. At the same time, mechanical and thermal properties are two critical factors that need to be considered for thermosetting polymer epoxy-based composites. In this paper, a commercial-grade butyl glycidyl ether was investigated as a modifier for promoting the low viscosity of the pre-polymer. The mechanical and thermal properties of cured composites with different modifier contents were evaluated, focusing especially on tensile strength and modulus, glass transition temperature, and fracture morphology. In addition, this study contributes to technical knowledge by identifying peculiarities of the cure behavior of the modified resins that may benefit the fabrication of composites containing fibrous and/or particulate fillers.

## 2. Materials and Methods

### 2.1. Materials

**Figure 1:**
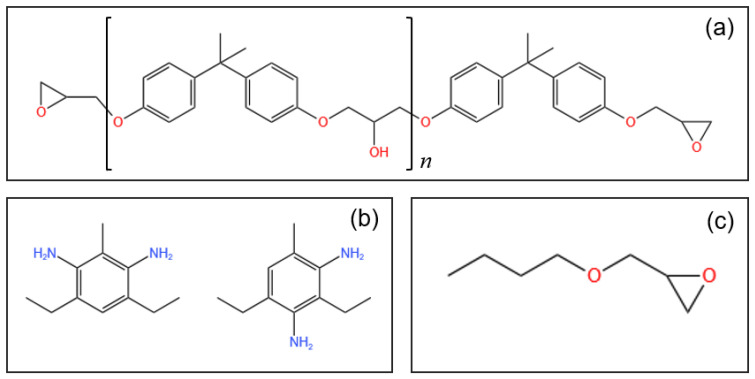
Chemical structures of (**a**) EPON826, (**b**) EPICURE-W, and (**c**) HELOXY 61.

In this study, the thermoset polymer base is the EPON826/EPICURE-W epoxy resin system (Miller-Stephenson), mixed at the weight ratio of 100:26.4. EPON826 is a DGEBA epoxy resin with an epoxide equivalent weight of 178–186, while EPICURE W is a methylene dianiline curative with a nitrogen content between 15.7% and 15.9%. The selected modifier is HELOXY 61 (Miller-Stephenson), which is a commercial-grade butyl glycidyl ether with an epoxide equivalent weight of 144–155. The chemical structures of EPON826, EPICURE-W, and HELOXY 61 are presented in Figure 1.

### 2.2. Material Fabrication

The resin base was first heated at 50 °C in an oven (Isotemp Oven, Fisher Scientific, Ottawa, ON, Canada) to lower its viscosity, benefiting the mixing process. Then, a stoichiometric amount of the curing agent and the modifier were introduced to the resin at room temperature and mixed at a speed of 500 rounds per minute for 5 min by a mechanical stirrer (Caframo, Georgian Bluffs, ON, Canada). The mixture was transferred into an open steel mold with an interior diameter of 100 mm, coated with a fluoroelastomer mold release (A373, Stoner, Quarryville, PA, USA), and cured at a temperature setting of 100 °C for 3 h until the mixture solidified into a solid circular disk. The oven was turned off and the cured disk was allowed to cool to room temperature. Afterwards, the cured sample was released and exposed to a post-cure cycle at 75 °C for 24 h. Five sample formulations containing different amounts of the modifier were fabricated, with mass fractions for the various constituent materials and formulation identifiers as listed in Table 1.

### 2.3. Material Characterization

Rheological properties of the EPON-0wt% and EPON-20wt% were evaluated using a parallel plate rheometer (Discovery HR-2, TA Instruments, New Castle, DE, USA) employing two different methods, i.e., isothermal conditions and ramped temperature. For the latter, heating started at room temperature and completed at 160 °C at a rate of 10 °C/min. Changes in rheological properties with respect to time were examined at an isothermal condition of 100 °C.

Thermal conditions during the curing of the various formulations were assessed at a constant ambient temperature using K-type thermocouples (operating temperature −50 °C to 700 °C and accuracy 0.75%). In this manner, the temperatures of the mixtures, i.e., a thermocouple, were immersed in a resin system and the ambient conditions inside the oven were monitored at a sampling rate of 1 min. Data were collected via an eight-channel data logger (OM-CP-OCTPRO, Omega, St-Eustache, QC, Canada). The Savitzky-Golay method in the OriginPro 2024b software (OriginLab, Northampton, MA, USA) was used to smooth the minor scatter of the recorded temperature data.

DSC tests were conducted with a DSC-Q1000 device (TA Instruments) to explore the curing behavior of the liquid polymer formulations and the glass transition temperature (*T*_g_) of cured materials. Tests were performed under nitrogen flow at a rate of 20 mL/min to maintain stable and consistent testing conditions. Recorded thermograms were processed with the TA Universal Analysis 2000 software (TA Instruments) to compute the heat flow. For the curing behavior analyses, the temperature was ramped from 20 °C to 300 °C at a rate of 5 °C/min. To determine *T*_g_, crushed powder with a sample mass of 3 mg to 5 mg was exposed to a constant temperature of 30 °C for 5 min and then heated to 300 °C at a rate of 20 °C/min. *T*_g_ was estimated as the inflection point of the stepwise transition in the thermogram.

The thermal stability of cured polymer formulations was evaluated by thermogravimetric analysis (TGA) using a TGA 5500 instrument (TA Instruments). The crushed powder of the polymer formulations was heated under a nitrogen atmosphere (flow rate of 25 mL/min) from room temperature to 600 °C at the ramp rate of 20 °C/min. A weight loss of 5% and 50% was used to characterize the decomposition behavior.

The thermo-mechanical behavior of cured epoxy samples was investigated by thermal-mechanical analysis (DMA) (model DMA 8000, Perkin-Elmer, Waltham, MA, USA). The in-plane sample dimensions for DMA testing were 15 mm by 5 mm, respectively. Sample thicknesses varied between 1.5 mm to 2.5 mm. Testing was performed in single cantilever bending mode. Measurements were taken over a temperature range from 26 °C to 150 °C with a heating rate of 5 °C/min. A cyclic deformation of 0.05 mm was applied at a constant frequency of 1 Hz. The damping factor was recorded as a function of temperature, allowing the glass transition temperature to be assessed as the peak of the recorded damping factor.

The stress-strain response of the cured samples was assessed using a universal testing machine (Model 5966, Instron, Norwood, MA, USA) according to the standard ASTM D638-14 [29]. Type V dogbone samples were prepared via waterjet cutting (type 2652, OMAX, Kent, MA, USA). Specimens were tested in quintuplicate. The tensile test speed was 1 mm/min. The strain was computed based on the elongation over the distance between the grips. The elastic modulus was estimated from the slope of the linear section of the stress-strain curves, i.e., a range between 0.0005 mm/mm and 0.0025 mm/mm was taken.

Fracture surfaces of failed specimens were probed using scanning electron microscopy (SEM). A field emission scanning electron microscope at an acceleration voltage of 10 kV was employed for this task (300 VP, Zeiss Sigma, Oberkochen, Germany). Prior to imaging, fracture surfaces were first coated with carbon, which was accomplished using a Leica EM SCD005 evaporative carbon coater (Wetzlar, Germany).

## 3. Results and Discussion

### 3.1. Rheological Characterization

**Figure 2:**
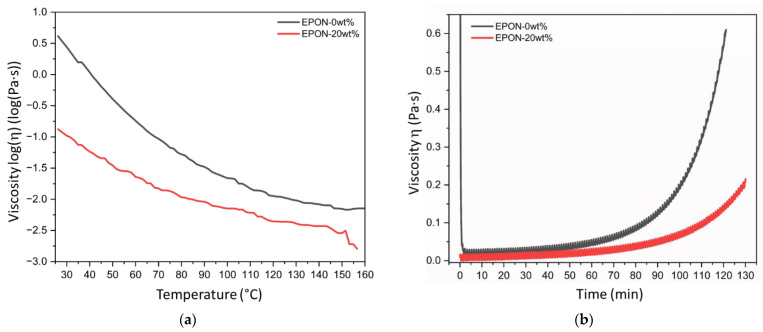
The viscosity of EPON-0wt% and EPON-20wt% for (**a**) increasing temperature, and (**b**) isothermal conditions at 100 °C.

Rheological characterizations were conducted with the formulations EPON-0wt% (pure epoxy resin) and EPON-20wt% epoxy resin (20 wt% modifier). Figure 2a depicts the measured viscosity with respect to temperature. (Note that the graph is plotted in a semi-log scale to depict differences at low viscosity values more clearly.) At room temperature, the 20 wt% modifier addition decreased the viscosity by 97% (i.e., EPON-0wt%: 4.12 Pa·s; EPON-20wt%: 0.13 Pa·s). It can be further observed that with rising temperature the difference in viscosity between the modified and unmodified resins decreased. Notably, at about 63 °C, EPON-0wt% had the same viscosity as EPON-20wt% at room temperature.

Figure 2b depicts viscosity versus time graphs for isothermal conditions at 100 °C. Since the resin systems were reactive, i.e., three-dimensional polymer networks formed during the curing process, viscosity is expected to increase with time. The results in Figure 2b revealed that retardation or possibly hindrance in the curing reaction occurred with the addition of the modifier to the epoxy resin system, which is similar to observations made in [20]. At the test start, the viscosity of the EPON-20wt% sample was considerably lower compared to EPON-0wt%, i.e., 0.01 Pa·s and 1.18 Pa·s, respectively. Referring to Table 2, the viscosity of the EPON-0wt% sample reached a minimum viscosity of 0.014 Pa·s in 4 min after the test start, upon which the viscosity increased in an exponential manner (see Figure 2b). In contrast, for the EPON-20wt% sample, the viscosity increased to the same value (0.014 Pa·s) in 40.6 min. The viscosity also continued to increase with time, yet at a lower rate than for the EPON-0wt% sample. Drawing attention again to the start of the test, the time difference between EPON-0wt% and EPON-20wt% to reach a viscosity of 0.014 Pa·s was 36.6 min, which can provide for extended processing time at low viscosity using the modified resin system, thus easing composites fabrication (e.g., providing time for mixing and material handling).

During the subsequent curing at the set temperature (i.e., 100 °C), the EPON-20wt% sample required a longer time to reach a given viscosity and thus a state of cure compared to the pure epoxy resin system. For example, 100.5 and 128.9 min were required to reach a viscosity of 0.20 Pa·s for EPON-0wt% and EPON-20wt%, respectively (see Table 2). Supposedly, the modifier causes a reduced rate of cure. The modifier contains equivalent reactive groups as the base resin (see Figure 1), which can react with the curative’s amine groups, thus diminishing the interactions between base resin and curative [30]. Note that the weight ratio of base resin to curative was fixed. Considering the base resin and curative as a ‘group’, the weight ratio of the ‘group’ to modifier decreases with higher modifier content, i.e., for EPON-10wt% and EPON-20wt%, the ratios were 9:1 and 4:1, respectively.

### 3.2. Curing Behavior for Various Epoxy Resin Systems

#### 3.2.1. Temporal Temperature Recordings in Samples during Isothermal Curing

**Figure 3:**
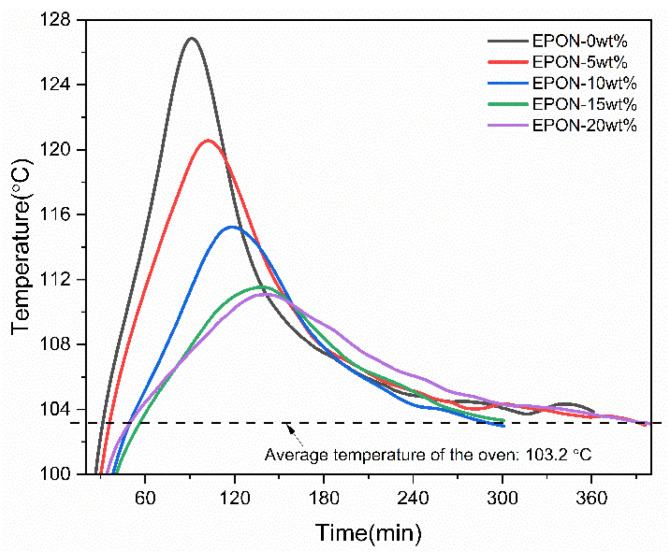
Temperature data versus time recorded during curing of various epoxy systems.

Figure 3 depicts temporal temperature recordings from thermocouples of the five epoxy systems during isothermal curing. The average ambient temperature at the sample location inside the oven was measured as 103.2 °C. During the curing process for each system, an ‘exothermal peak’ was observed, caused by covalent bonds forming at reactive sites of the resin system, thus releasing heat. Notably, the increase of the modifier content resulted in a wider exothermal peak with reduced magnitude and a longer time to reach the maximum temperature.

Table 3 shows the average temperatures computed for durations spanning from the test start (at room temperature) to the point upon which the resin formulations reached the oven temperature after passing the peak point. It can be observed that average temperatures over this duration decreased with modifier loading, i.e., 106.9 °C and 104.0 °C for the epoxy system without and with 20 wt% modifier content, respectively. Table 3 also lists the times for the resin formulations to reach (i) the oven temperature initially and (ii) the peak point (maximum temperature). For (i), samples took longer to reach the oven temperature with rising modifier content, e.g., 31.5 min and 50.5 min for EPON-0wt% and EPON-20wt%, respectively. It is understood that for this initial phase, heat supplied by the oven significantly contributes to the rise in temperature, whereas heat released by exothermic polymerization is responsible for the subsequent temperature increase. The peak temperatures, ranging from 127.3 °C to 111.2 °C, were reached in 89 min and 142 min for the pure epoxy system and the formulation with the highest modifier content, respectively. These data are consistent with the aforementioned attenuating effect that the modifier has on the resin system’s cure reaction.

#### 3.2.2. DSC Ramped Temperature Experiments with Different Resin Formulations

**Figure 4:**
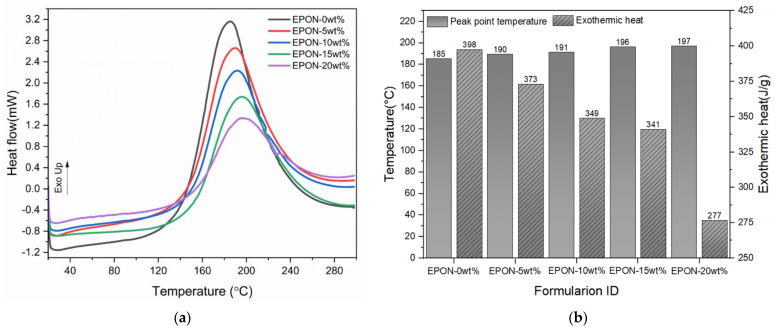
Cure kinetics of different epoxy formulations: (**a**) DSC thermograms showing heat flow versus temperature, and (**b**) reduced data for maximum temperature and specific exothermic heat.

The DSC thermograms depicted in Figure 4a provide additional information about the cure kinetics of the different resin formulations during curing at rising ambient temperature. The graph reveals changes in heat flow for the different formulations. Specifically, the heat flow at the peak point decreased substantially with the increase of modifier content while the temperature at which the peak point was reached increased modestly. The reduced thermogram data in Figure 4b shows that between the resin formulations with zero and 20 wt% modifier content, the peak point temperature increased from 185 °C to 197 °C while the exothermic heat decreased from 398 J/g to 277 J/g (a change of 6.5% and 30.4%, respectively). Given the fixed rate of temperature increase for these tests, the rise in peak point temperature is synonymous with a longer time to reach the peak. Also, considering the observed drop in exothermic heat, these results are indicative of a cure reaction retardation or hindrance that the modifier addition imparted on the resin system. Hence, the findings from the DSC experiments are consistent with the observations made in the rheological analysis and the temporal temperature study when curing the formulations at isothermal conditions.

### 3.3. Glass Transition Temperature and Thermal Stability of Cured Resin Formulations

**Figure 5:**
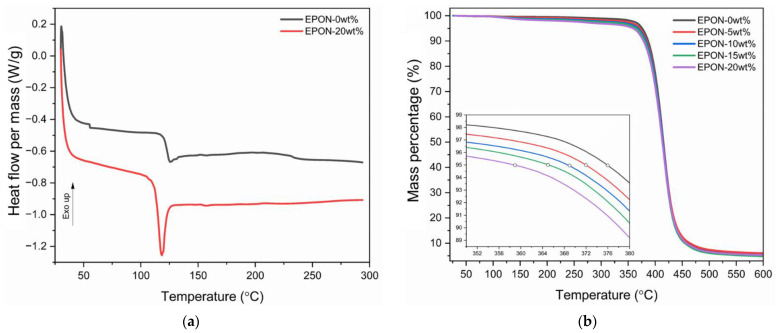
Thermograms of cured epoxy formulations: (**a**) DSC and (**b**) TGA.

The DSC thermogram in Figure 5a depicts the data collected for cured resin formulations EPON-0wt% and EPON-20wt%. (Note that the remaining formulations were omitted for clarity.) In the case of the pure resin system, the curve exhibits an endothermic drop at a temperature of 122.5 °C, which is interpreted as the glass transition of the pure resin system. For the resin system containing 20 wt% modifier, a notable endothermic transition, mimicking a melting event, at the glass transition can be observed (Note that the other resin formulation containing the modifier exhibited similar behavior). It is inferred that this ‘apparent melting’ behavior relates to molecular relaxation through the glass transition [31]. However, further analysis is required to corroborate this supposition (using e.g., DSC analysis after annealing the material above *T*_g_, see [31]), which is beyond the scope of this study. Considering the initial inflection point for the EPON-20wt% formulation, a slight drop in *T*_g_ was ascertained compared to the neat epoxy system. A similar behavior was reported in [20]. Corresponding data for the five resin formulations is summarized in Table 4. Subsequent to the heat flow events at the glass transition temperature, the curves describe a near-constant behavior for rising temperatures, which suggests that the resin systems were fully cured during the described fabrication process.

The thermal stability of the cured resin formulations was investigated by determining decomposition temperatures via TGA. The temperature at the start of decomposition was estimated as the point of the 5% mass loss [32]. The graph shown in Figure 5b for the five cured resin formulations reveals nearly identical degradation behavior featuring a single degradation stage [33]. Referring to the inset in Figure 5b, temperatures at 5% mass loss are seen to decrease slightly with rising modifier content. Further details on the decomposition temperatures for a mass loss of 5% and 50% are listed in Table 4. For example, the decomposition temperatures at 5% mass loss were 376 °C and 359 °C for the EPON-0wt% and EPON-20wt% samples, respectively. For a mass loss of 50%, temperatures were practically identical as differences were less than 1%. Presumably, changes in polymer morphology, such as a lower crosslinking density, may have affected the decomposition behavior slightly at its onset but not in an advanced decomposition stage. Nevertheless, overall, it can be ascertained that the modifier addition did not appreciably affect the thermal stability of the epoxy systems.

### 3.4. Glass Transition Temperature Measurement by Thermal-Mechanical Testing

Thermal-mechanical testing was employed to further characterize the glass transition behavior of the five cured epoxy formulations. When associating the peak of the recorded damping factor (tan delta) with *T*_g_, a declining trend in *T*_g_ values was determined that is similar to the DSC measurements, albeit with higher values for lower modifier contents. As shown in Table 4, *T*_g_ values for the EPON-20wt% samples are practically identical whereas for the pure resin system, *T*_g_ values vary by about 6% between the different testing methods. Nevertheless, the DMA testing confirms *T*_g_ values that lie considerably above the initial curing temperature of 100 °C, as well as a declining trend in *T*_g_ with increasing modifier content.

### 3.5. Tensile Properties and Fracture Surface of Cured Epoxy Composites

**Figure 6:**
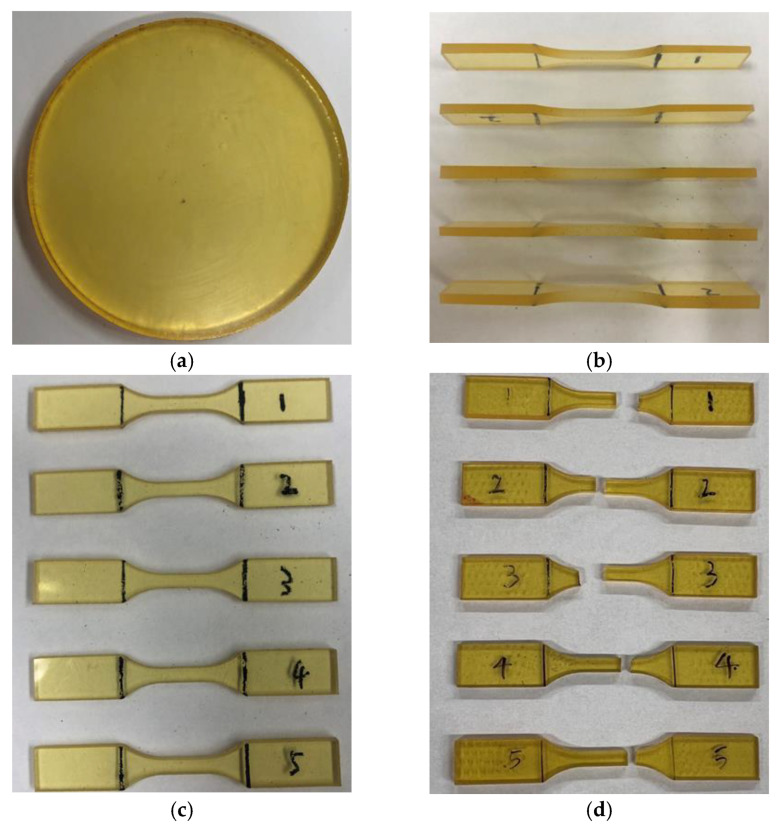
Photographs of (**a**) cured epoxy resin disk, (**b**) side view in through-thickness direction of tensile samples, and top surfaces of tensile samples (**c**) before and (**d**) after tensile failure.

Figure 6 shows an example of a cured epoxy resin disk and five dogbone-shaped samples from side and top views. No obvious imperfections, such as unevenness or voids, were observed. Hence, the quality of the specimens was deemed adequate for tensile testing. In Figure 6d, specimens are shown after experiencing tensile failure. It can be observed that failure consistently occurred within the specimen gauge section, validating the chosen specimen preparation and testing methodology.

**Figure 7:**
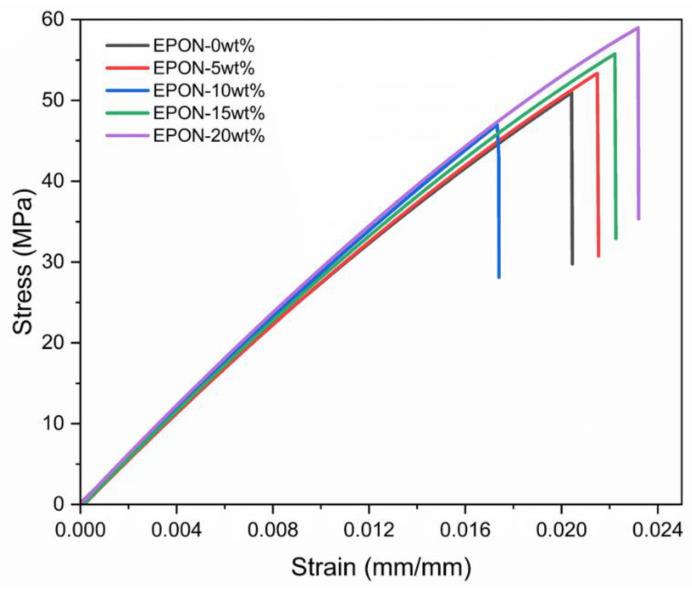
Typical stress-strain curves of cured epoxy formulations.

The typical stress-strain behavior for the five cured epoxy formulations is depicted by the graphs in Figure 7. The slopes of the different stress-strain curves are quite consistent and appear nearly congruent in the initial strain range, e.g., up to 0.004 mm/mm. However, differences can be observed to emerge for higher strains, which are attributed to modest mechanical property changes related to the different modifier contents. Irrespective of their modifier content, all specimens exhibited brittle fracture, which inherently leads to comparatively large scatter in terms of failure strength.

**Figure 8:**
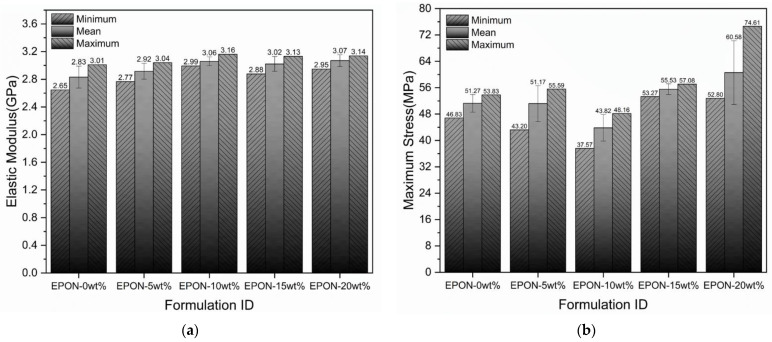
Reduced data from tensile testing: (**a**) elastic modulus, and (**b**) tensile strength. Error bars indicate standard deviations from the mean.

The slope of the stress-strain curves in the strain range from 0.0005 mm/mm to 0.0025 mm/mm was utilized to estimate the elastic moduli of the tested samples. Reduced data for modulus and strength are summarized in Figure 8a and 8b, respectively. The graphs shown in Figure 8a indicate a minor influence of the modifier content on the elastic response of the cured epoxy formulations. The elastic modulus describes an increase trending with the increase of the modifier content. For instance, the computed elastic modulus was 2.83 GPa and 3.07 GPa for EPON-0wt% and EPON-20wt% with small standard deviations of 0.26 GPa and 0.08 GPa, respectively. The addition of the 20 wt% modifier thus raised the modulus by 8.5%, indicating that the modifier has a moderately positive influence on raising the elastic modulus. It is worth mentioning that the elastic modulus for the neat resin system, as specified by the material supplier, is 2.48 GPa. The somewhat higher values obtained in the present analysis are seen to be a consequence of the employed testing methodology (i.e., using stroke data from the testing machine to calculate strain rather than an extensometer). Nevertheless, for the purpose of comparing the different resin formulations, the employed testing methods are considered adequate.

In terms of the maximum stress at failure, it can be observed that modifier addition also resulted in increased values compared to the neat epoxy system, as shown in Figure 8b. The increase in average maximum stress between the EPON-0wt% and EPON-20wt% is quantified as 18.2%. However, as mentioned above, significant data scatter indicated by high standard deviations can be observed for all epoxy formulations due to the brittle nature of the materials. Strength data must therefore be considered with lower confidence.

**Figure 9:**
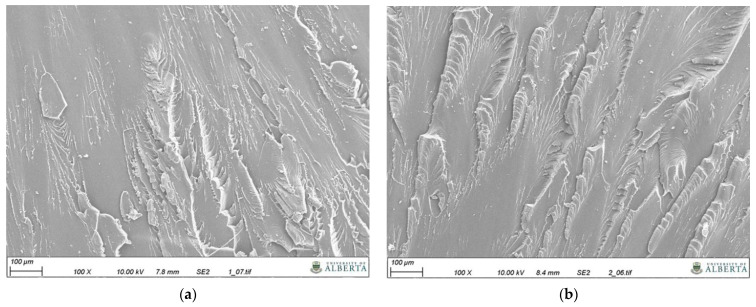

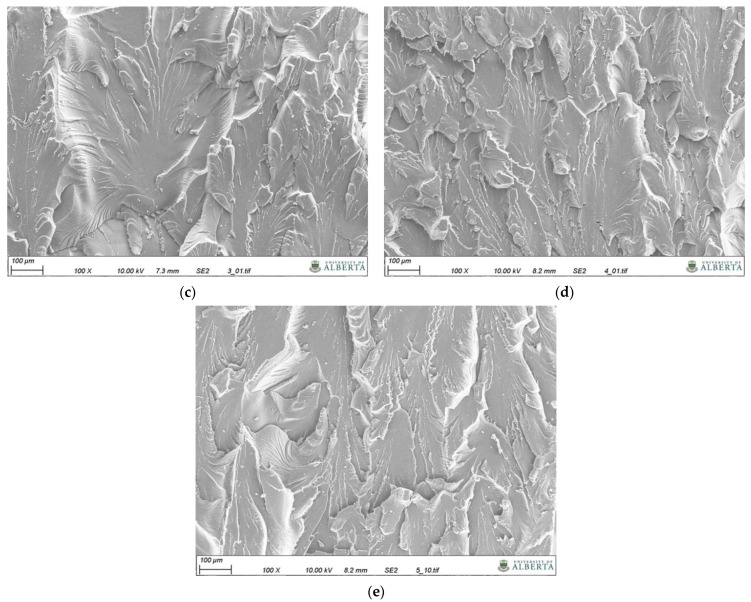
Representative fracture surface micrographs of failed epoxy specimens: (**a**) EPON-0wt%, (**b**) EPON-5wt%, (**c**) EPON-10wt%, (**d**) EPON-15wt%, and (**e**) EPON-20wt%.

The morphology of fracture surfaces of failed specimens was investigated using SEM imaging. Sample micrographs are shown in Figure 9. Fracture surfaces were observed to be richer in features, i.e., having a higher surface roughness, with increasing modifier content. For instance, the micrographs for an EPON-0wt% specimen in Figure 9a exhibit a rather smooth surface with fracture features of limited quantity and magnitude. Conversely, the micrograph for an EPON-20wt% in Figure 9e displays a much rougher surface topology. Since in general, an increase in surface roughness is indicative of higher toughness of the epoxy material [20], the present observations agree with the ascertained trend of material strength increasing with the rise of modifier content. The authors postulate that changes in polymer chain morphology caused by modifier addition resulted in improved fracture resistance. Still, additional research is needed to fully appreciate the effect of the modifier on the fracture behavior and strength of the fabricated epoxy formulations.

## 4. Conclusions

In this study, the effects of a rheological additive (butyl glycidyl ether) on the rheological, thermal, and mechanical properties of a common epoxy system (diglycidyl ether of bisphenol A with a methylene dianiline curative) was investigated. The modifier was added at four different weight percentages to the base resin system, i.e., 5 wt%, 10 wt%, 15 wt%, and 20 wt%. Material samples were successfully fabricated from the pure and modified epoxy systems by curing them at 100 °C, followed by a post-cure at 75 °C. Rheological analyses revealed that a 20 wt% modifier addition reduced the prepolymer viscosity by 97% compared to the base resin system at room temperature. Studying the rheology as well as monitoring thermal conditions during curing consistently revealed an attenuating effect that the modifier had on the resin system’s cure reaction. That is, the onset of the cure reaction was delayed, providing a substantially longer pot life and processing window to a modified resin system (in the order of tens of minutes), which can be beneficial to some applications. In addition to delaying the onset of cure, the modifier reduced the exothermic reaction during cure, leading to an extended exothermic event yet with lower peak temperatures. The peak temperature was reduced by approximately 13% for the highest modifier loading.

The thermal characteristics of the various cured resin formulations were examined using differential scanning calorimetry, thermogravimetric analysis, and dynamic mechanical analysis. These studies showed that the glass transition temperature of the resin formulations was notably above the curing temperature. Compared to the neat resin system, the addition of the modifier caused the glass transition temperature to drop moderately, for example, by 5% to 11%, depending on the analysis technique, for the highest modifier content. Conversely, the addition of the modifier had a negligible influence on the resin systems’ degradation behavior at high temperatures.

Mechanical testing showed that adding the rheological modifier to the base resin led to a modest rise in elastic modulus and strength at break. For the highest modifier loading, the elastic modulus rose on average by 8.5%. In terms of strength, the addition of the modifier seems to be beneficial. For instance, strength increased by about 18% for the highest modifier content. However, the brittle nature of the fabricated materials caused a large scatter in values, and hence, the effect of the modifier on strength cannot be fully appreciated through the completed experiments.

Overall, the study demonstrated that the addition of the modifier to the base epoxy resin is beneficial primarily for the processing stage when reduced viscosity is needed to create composite materials, such as those containing fibrous and/or particulate fillers. The effects of the modifier on the materials’ thermal and mechanical properties are benign even at a comparatively high modifier ratio of 20 wt%.

## Figures and Tables

**Table 1:** Mass fractions of constituent materials and identifiers for the different sample formulations.

Formulation ID	EPON-0wt%	EPON-5wt%	EPON-10wt%	EPON-15wt%	EPON-20wt%
EPON 826	79.11	75.16	71.20	67.25	63.29
EPICURE W	20.89	19.84	18.80	17.75	16.71
HELOXY 61	0	5	10	15	20

**Table 2:** Reduced data of viscosity with time from isothermal condition at 100 °C.

Viscosity of Sample (Pa·s)	Time to Reach Viscosity from Test Start (Minutes)	
	EPON-0wt%	EPON-20wt%
0.014	4.0	40.6
0.05	63.2	89.9
0.10	83.8	110.8
0.15	94.3	121.3
0.20	100.5	128.9

**Table 3:** Reduced data from temporal temperature recordings for different resin systems.

Formulation ID	EPON-0wt%	EPON-5wt%	EPON-10wt%	EPON-15wt%	EPON-20wt%
Average temperature during curing (°C)	106.9	105.8	104.1	103.4	104.0
Time to reach oven temperature (min)	31.5	36.5	49.5	57.8	50.5
Peak point temperature (°C)	127.3	120.8	115.3	111.6	111.2
Time to reach peak temperature (min)	89	102	119	133	142

**Table 4:** Glass transition temperatures *T*_g_ assessed by DSC and DMA, and decomposition temperatures measured by TGA for cured resin formulations.

Formulation ID	*T*_g_ (°C)		Decomposition Temperature (°C)	
	By DSC	By DMA	At 5% Mass Loss	At 50% Mass Loss
EPON-0wt%	122.5	130.8	376	415
EPON-5wt%	119.7	128.8	372	415
EPON-10wt%	119.1	125.9	369	414
EPON-15wt%	118.3	121.7	365	413
EPON-20wt%	116.2	116.1	359	413

## Data Availability

The original contributions presented in the study are included in the article, further inquiries can be directed to the corresponding author.
